# Unique fluorophilic pores engineering within porous aromatic frameworks for trace perfluorooctanoic acid removal

**DOI:** 10.1093/nsr/nwad191

**Published:** 2023-07-10

**Authors:** Chi Zhang, Junchao Dong, Panpan Zhang, Lei Sun, Liu Yang, Wenjian Wang, Xiaoqin Zou, Yunning Chen, Qingkun Shang, Danyang Feng, Guangshan Zhu

**Affiliations:** Faculty of Chemistry, Northeast Normal University, Changchun130024, China; Faculty of Chemistry, Northeast Normal University, Changchun130024, China; Faculty of Chemistry, Northeast Normal University, Changchun130024, China; Institute of Molecular Sciences and Engineering, Institute of Frontier and Interdisciplinary Science, Shandong University, Qingdao266237, China; Faculty of Chemistry, Northeast Normal University, Changchun130024, China; Faculty of Chemistry, Northeast Normal University, Changchun130024, China; Faculty of Chemistry, Northeast Normal University, Changchun130024, China; Faculty of Chemistry, Northeast Normal University, Changchun130024, China; Faculty of Chemistry, Northeast Normal University, Changchun130024, China; Faculty of Chemistry, Northeast Normal University, Changchun130024, China; Faculty of Chemistry, Northeast Normal University, Changchun130024, China

**Keywords:** porous aromatic frameworks, fluorophilicity, perfluorooctanoic acid, adsorption removal, halogen interactions

## Abstract

Perfluorooctanoic acid (PFOA), a representative of per/polyfluorinated alkyl substances, has become a persistent water pollutant of widespread concern due to its biological toxicity and refractory property. In this work, we design and synthesize two porous aromatic frameworks (PAF) of PAF-CF_3_ and PAF-C_2_F_5_ using fluorine-containing alkyl based monomers in tetrahedral geometry. Both PAFs exhibit nanosized pores (∼1.0 nm) of high surface areas (over 800 m^2^ g^−1^) and good fluorophilicity. Remarkable adsorption capacity (˃740 mg g^−1^) and superior efficiency (˃24 g mg^−1^ h^−1^) are achieved toward the removal of PFOA with 1 μg L^−1^ concentration owing to unique C–F···F–C interactions. In particular, PAF-CF_3_ and PAF-C_2_F_5_ are able to reduce the PFOA concentration in water to 37.9 ng L^−1^ and 43.3 ng L^−1^, below EPA regulations (70 ng L^−1^). The reusability and high efficiency give both PAFs a great potential for sewage treatment.

## Teaser

Two porous aromatic frameworks containing perfluoroalkyl groups are designed and successfully synthesized with good fluorophilicity and high porosity. They efficiently remove PFOA below regulation limit, candidating them for water purification.

## INTRODUCTION

Perfluorooctanoic acid (PFOA), one of the most typical perfluorinated compounds, is widely used in fire-fighting foam, and in the lubricant and electroplating industries, owing to its physicochemical properties of high thermal/chemical stability, amphiphilicity and surfactant ability [1,2]. However, the high energy of the C–F bond makes PFOA a bioaccumulative, a ubiquitously distributed and persistent organic pollutant to the water system; and it is difficult to be completely removed by water treatment plants [3]. Investigations have confirmed that the accumulation of low-content PFOA in an organism can cause toxicity to hepatic, mammary gland, immune and endocrine systems [4,5]. A lifetime drinking water health advisory for PFOA of 70 ng L^−1^ is promulgated to be the upper limit by the environmental protection agency (EPA) of the United States (2016) [6].

Technologies, including adsorption, electrochemical oxidation, coagulation and ultraviolet/ultrasonic irradiation have been practically applied to mitigate PFOA pollution in water bodies [7–9]. Among them, the adsorption approach is considered the most economical and effective means because other chemical treatments are subjected to energy penalties and operational difficulties that are a consequence of the high chemical stability of PFOA [10]. Promising adsorbents for PFOA removal can be categorized as activated carbons [11,12], resins [13], minerals [14], biomaterials [15], and carbonaceous/polymeric hybrids [16,17]. Activated carbons (ACs), including granular activated carbon (GAC) and powdered activated carbon (PAC), have high adsorption capacities and can be produced at low cost, thus they are widely used for PFOA removal. Nevertheless, the low efficiency of PFOA at environmentally relevant concentrations together with regeneration difficulty restricts ACs’ further application [18]. Apparently, a trade-off problem between capacity and adsorption rate cannot be neglected in both porous and nonporous materials [19]. Porous materials such as ACs usually have high adsorption capacities, and concurrently they have quite low adsorption rates ascribed to slow intraparticle diffusion. Vice versa, rapid attainment of equilibrium occurs in nonporous materials, such as functionalized polymers; however, their adsorption capacities are considerably lower. Therefore, the designed synthesis of a novel adsorbent that possesses both high capacity and fast adsorption rate is challenging.

Porous organic frameworks (POFs) combine high porosity from ACs and facile functionalization from polymers; they are a platform to potentially solve the aforementioned trade-off problem. As to PFOA, ionic, amino and alkyl groups can attract the PFOA molecule via electrostatic interaction, hydrogen bonding and hydrophobic effect [10,19–22]; and some efforts were invested to graft functional groups on different POFs for affinity improvement in order to remove PFOA, with the electrostatic interaction prevailing among adsorption mechanisms [20,23]. A recent theoretical study indicates that C–F···F–C interaction with energy of 1–20 kcal mol^−1^ is strong enough to achieve the specific adsorption by fluorine-containing adsorbents [24]. Besides, the designed adsorbents on the basis of this interaction, in contrast to other adsorption mechanisms, are insusceptible to water environments such as pH, ion strength, coexistent natural organic matter, etc. [10,19,25,26]. Thus, this principle can be tentatively adopted for PFOA removal; but so far, only a few POFs have been designed or functionalized with fluorine [18]. Porous aromatic frameworks (PAFs), as a representative of POFs, are composed of abundant benzene rings linked by C–C covalent bonds which are formed through a variety of coupling reactions [27]. PAFs, characteristic of uniform pores, high surface areas and framework diversification, are ideal platforms for the development of capacious and functional adsorbents; and they are of great potential in the adsorption of gases [28], metal ions [29], dyes [30] and contaminants [31,32]. To this regard, C–F bonded PAFs with proliferated chemistry are worthy of particular investigation for the application of PFOA removal.

In this study, we constructed two perfluoroalkyl-functionalized PAFs from the perspective of C–F···F–C interaction, named PAF-CF_3_ and PAF-C_2_F_5_, through a bottom-up strategy, for the efficient removal of PFOA from water. Tetrahedral building units were adopted to yield open frameworks with high surface areas. The bottom-up synthesis ensured complete and molecularly uniform distribution of CF_3_ (C_2_F_5_) in the framework, in contrast to post-synthesis. Introduced CF_3_ and C_2_F_5_ functional groups enhanced the affinity for PFOA via fluorophilicity. The high performance of PAF-CF_3_ and PAF-C_2_F_5_ for PFOA removal from contaminated water was well demonstrated.

## RESULTS AND DISCUSSION

**Figure 1.**
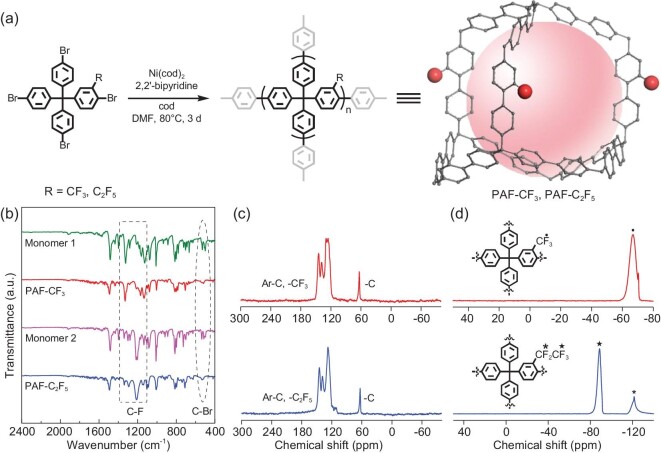
(a) Synthetic routes of PAF-CF_3_ and PAF-C_2_F_5_; (b) infrared (IR) spectra of as-synthesized monomer 1 ((4-bromo-3-trifluoromethylphenyl)tris(4-bromophenyl)methane), monomer 2 ((4-bromo-3-pentafluoroethylphenyl)tris(4-bromophenyl)methane), PAF-CF_3_ and PAF-C_2_F_5_; (c) ^13^C and (d) ^19^F NMR spectra of PAF-CF_3_ (red) and PAF-C_2_F_5_ (blue).

–CF_3_ and –C_2_F_5_, as perfluoroalkyl groups, were selected to increase the fluorophilic properties of the frameworks according to C–F···F–C interaction. The target materials of PAF-CF_3_ and PAF-C_2_F_5_ were synthesized using Ni(0)-catalyzed Yamamoto type Ullmann reactions (Fig. 1a). In the infrared (IR) spectra (Fig. 1b), the C–Br vibration associated bands (∼500 cm^−1^, indicated in dashed ellipse) are not present in PAF-CF_3_ and PAF-C_2_F_5_, confirming the completeness of the coupling reaction. The corresponding C–F IR bands at 1150–1350 cm^−1^ (indicated in dashed rectangle) are observed, showing the existence of perfluoroalkyl groups in both PAF materials. ^13^C and ^19^F solid-state nuclear magnetic resonance (NMR) (Fig. 1c and d) spectroscopies were used to study the chemical environments of different atoms within PAF-CF_3_ and PAF-C_2_F_5_. In theory, there are 12 and 13 types of carbons in different chemical environments in PAF-CF_3_ and PAF-C_2_F_5_, respectively. The chemical shifts of carbon on the benzene rings lie between 110 and150 ppm, and they overlap the signals that associated to the carbon atoms of CF_2_ and CF_3_ (Fig. 1c). ^19^F NMR spectra confirm the existence of CF_3_ and C_2_F_5_ in the skeletons of PAF-CF_3_ and PAF-C_2_F_5_ (Fig. 1d).

The morphological characteristics of PAF-CF_3_ and PAF-C_2_F_5_ were determined by scanning electron microscopy (SEM) (Supplementary Fig. 3, in the online supplementary file). Both PAFs appear as aggregation of small spherical particles (Supplementary Fig. 3c and g), and such morphology is similar to other reported PAF materials [33]. The uniform distributions of fluorine in PAF-CF_3_ and PAF-C_2_F_5_ can be seen from the images of elemental mapping (Supplementary Fig. 3b and f), benefited from the bottom-up synthesis. Moreover, energy-dispersive X-ray (EDX) spectra (Supplementary Fig. 4) indicate that the C/F ratios are near the theoretical values (Supplementary Table 1). The water contact angles of PAF-CF_3_ and PAF-C_2_F_5_ are 133.2° and 145.8°, respectively (insets in Supplementary Fig. 3d and h), indicating the hydrophobic properties of PAF particles. In addition, water adsorption isotherms convex to the pressure axis and low initial uptakes indicate that PAF pores are hydrophobic (Supplementary Fig. 3d and h). The hydrophobic properties of PAFs can be correlated with the presence of fluorine atoms in the frameworks [34]. The powder X-ray diffraction (PXRD) data indicate that the two PAFs are amorphous (Supplementary Fig. 5). The thermogravimetric analysis curves (Supplementary Fig. 6) show that apparent weight losses of PAF-CF_3_ and PAF-C_2_F_5_ occur after ∼520°C, demonstrating their high thermal stabilities due to high C–F bond energy (∼485 kJ mol^−1^) [35].

The porous structures of PAF-CF_3_ and PAF-C_2_F_5_ were investigated by N_2_ physisorption. The isotherms at 77 K show rapid N_2_ adsorptions at low pressures and gradual increases with growing pressure (Supplementary Fig. 7a and c), illustrating that both PAFs are typical microporous materials. The hysteresis between adsorption and desorption is possibly caused by N_2_ cavitation in ink-bottle pores of PAFs. The surface areas for PAF-CF_3_ and PAF-C_2_F_5_ are 972.8 m^2^ g^−1^ and 809.8 m^2^ g^−1^, respectively, which were calculated from the N_2_ adsorption isotherms using the Brunauer-Emmett-Teller (BET) model. The major pore sizes of PAF-CF_3_ and PAF-C_2_F_5_ were quantified to be 12 Å and 10 Å (Supplementary Fig. 7b and d), respectively, according to nonlocal density functional theory (NL-DFT), matching the PFOA molecule (∼1.2 nm); suggesting that both PAFs are good candidates for hosting PFOA.

**Figure 2.**
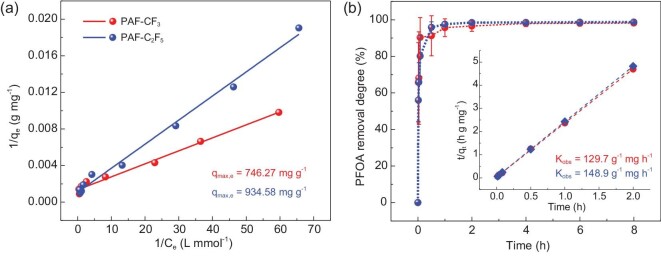
(a) Adsorption isotherms of PAF-CF_3_ and PAF-C_2_F_5_ for PFOA and the Langmuir fits (PAF dosage: 200 mg L^−1^, [PFOA]_0_ = 10-1000 mg L^−1^), and (b) kinetics of PFOA (200 μg L^−1^) adsorption on PAF-CF_3_ and PAF-C_2_F_5_ (PAF dosage: 500 mg L^−1^), inset in (b) is the corresponding pseudo-second-order plots.

The PFOA adsorption of PAF-CF_3_ and PAF-C_2_F_5_ was performed in aqueous solution at ambient condition (∼298 K, atmospheric pressure). For comparison, archetypal PAF-1 without fluorine groups was also synthesized and tested using the same experimental procedure. The PFOA adsorption isotherms were obtained from [PFOA]_0_ = 10-1000 mg L^−1^ using [PAF] = 200 mg L^−1^ in 48 h. According to the Langmuir model (Fig. 2a), PAF-CF_3_ and PAF-C_2_F_5_ have maximum adsorption capacities of 746.27 mg g^−1^ and 934.58 mg g^−1^, respectively. Higher capacity is observed for PAF-C_2_F_5_ due to its higher fluorine content and stronger hydrophobicity although PAF-CF_3_ and PAF-C_2_F_5_ have comparable surface areas. The maximum adsorption capacity of PAF-1 is 1888.97 mg g^−1^ (Supplementary Fig. 9), which is attributed to its extraordinary large surface area (4638.8 m^2^ g^−1^, Supplementary Fig. 8). Literature shows that PAF-CF_3_ and PAF-C_2_F_5_ have impressive PFOA adsorption capacities (Supplementary Table 2), outperforming most adsorbents such as ACs, metal-organic frameworks (MOFs), biochars and porous organic polymers [10,12,14,16,18–20,36–50]. To be noted, the K_L_ values (Supplementary Table 2) measuring the affinity coefficient for PAF-CF_3_ and PAF-C_2_F_5_ are much higher than that of the non-fluorinated PAF-1, revealing the positive effect of the C−F···F−C interaction for PFOA adsorption. According to the parameter of n related to the adsorption strength (1/n was obtained from Freundlich model fitting in Supplementary Fig. 10), the introduction of fluorine-containing functional groups indeed made the adsorption process more favorable with PAF-CF_3_ and PAF-C_2_F_5_ compared to PAF-1. The adsorption isotherm in Supplementary Fig. 11 shows that the adsorption capacity of PAF-1 is higher than those of PAF-CF_3_ and PAF-C_2_F_5_ at a higher initial PFOA concentration (˃500 mg L^−1^), the results are obviously reversed at lower initial concentration (˂200 mg L^−1^) which is normally detected from the water system. We speculate that the decisive factor that determines the adsorption capacity is PAF surface area when PFOA is at a high concentration. In contrast, under the condition of low concentration of PFOA, fluorophilic effect becomes dominant because the F-binding site can strongly attract PFOA into PAF-CF_3_ (PAF-C_2_F_5_) via the C − F···F − C interaction.

**Figure 3.**
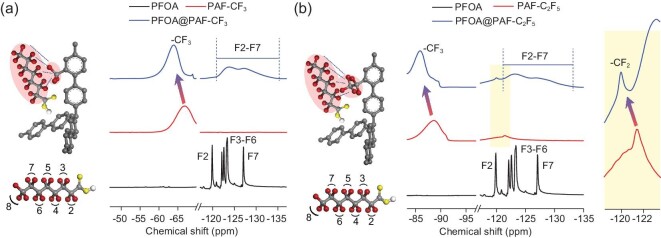
Solid state ^19^F NMR spectra of PFOA, PAF-CF_3_ and PFOA@PAF-CF_3_ (a), and PAF-C_2_F_5_ and PFOA@PAF-C_2_F_5_ (b).

Further, the interaction between PAF and PFOA was investigated by density functional theory (DFT). Fragments of tetraphenylmethane and hydrofluoroalkane-substituted tetraphenylmethane were chosen as the computational models. As shown in Supplementary Fig. 12, the interaction region indicators suggest that the van der Waals forces widely exist between the PFOA molecule and phenyl rings of all PAFs [51,52]. It is worth noting that halogen bonding appears between the F atom in molecular chain of PFOA and F atom in fluoroalkyl group grafted to the PAF framework, confirming the existence of C−F···F−C interaction. Hydrogen bonding of -OH in COOH with –F in CF_3_ (C_2_F_5_) also participates in the interaction of PFOA with PAF. Halogen and hydrogen bonds account for the higher adsorption capability of PAF-CF_3_ and PAF-C_2_F_5_ than that of PAF-1 at low PFOA concentrations, in accordance with more negative binding energies (−9.43 kcal mol^−1^ and −9.56 kcal mol^−1^ for PAF-CF_3_ and PAF-C_2_F_5_, −5.48 kcal mol^−1^ for PAF-1). Denser fluorine in PAF-C_2_F_5_ contributes to the shorter F···H distance (2.04 Å) than PAF-CF_3_ (2.08 Å), supporting the stronger affinity of PAF-C_2_F_5_ with PFOA. The solid state ^19^F NMR spectra of PFOA@PAFs were practically measured (Fig. 3). The comparisons of PFOA@PAFs with initial PAFs show that CF_3_ in PAFs have apparently different chemical shifts: −66.7 ppm in PAF-CF_3_ and −63.9 ppm in PFOA@PAF-CF_3_, −88.6 ppm in PAF-C_2_F_5_ and −85.9 ppm in PFOA@PAF-C_2_F_5_. Correspondingly, the signals of F2–F7 from PFOA are integrally shifting in an opposite direction. These variations are presumably generated from the C−F···F−C interaction between PFOA and framework. It is obvious that the introduction of fluorinated-functional groups to PAFs plays a positive effect on PFOA adsorption.

The adsorption rate is also a very important determinant for PFOA removal efficiency. The kinetics of PFOA adsorption were followed on PAF-1, PAF-CF_3_ and PAF-C_2_F_5_ (PAF use is 500 mg L^−1^, initial concentration of PFOA is 200 μg L^−1^). The results in Fig. 2b show that PAF-CF_3_ and PAF-C_2_F_5_ acquire fast PFOA adsorption with initial rates of 23.9 mg g^−1^ h^−1^ and 26.0 mg g^−1^ h^−1^, respectively. Removal degrees reached over 95% on both fluoroalkyl-functionalized PAFs within a short time period of 1 h, such a high value was maintained over a long time period (up to 8 h). A comparably low removal degree of 59.4% is obtained from PAF-1 under the same condition (Supplementary Fig. 13), which is due to its lack of functional group for PFOA. Time-dependent data were subsequently quantified by Ho and McKay's pseudo-second-order adsorption model which is widely used to describe PFOA adsorption kinetics [14,18,20,37,46]. The higher goodness of fit shown in Supplementary Fig. 14 suggests that the pseudo second order is an appropriate model instead of the pseudo first order. The values for parameter K_obs_ which is used to measure the rate of the adsorbent reaching adsorption equilibrium are calculated to be 129.7 g mg^−1^ h^−1^ and 148.9 g mg^−1^ h^−1^ for PAF-CF_3_ and PAF-C_2_F_5_ (the inset of Fig. 2b), respectively. The large K_obs_ of PAF-CF_3_ and PAF-C_2_F_5_ position them in the top rank of all reported materials (Supplementary Table 3). N_2_ adsorption-desorption isotherms of PAF-CF_3_ and PAF-C_2_F_5_ after PFOA adsorption (Supplementary Fig. 15 and Fig. 16) were acquired for calculation of BET surface area, pore size and micropore volume. All these parameters are significantly reduced after PFOA adsorption (Supplementary Table 4), evidencing that the adsorption process mainly takes place in the inner pore rather than on the outer surface.

**Figure 4.**
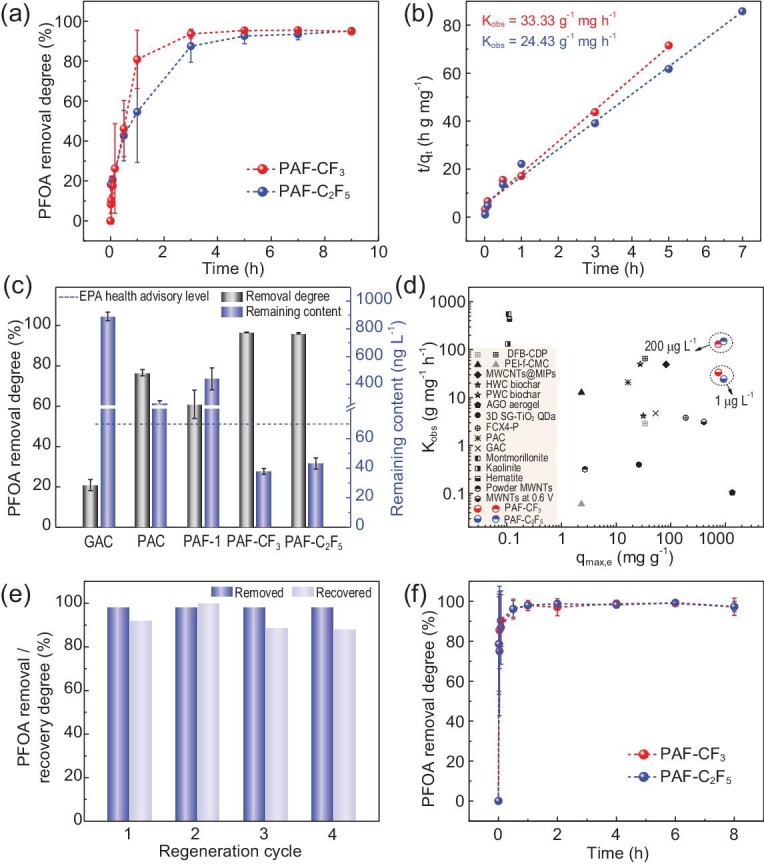
Kinetics of PFOA (1 μg L^−1^) adsorption (a) and pseudo-second-order plots (b) of PAF-CF_3_ and PAF-C_2_F_5_ (PAF dosage: 10 mg L^−1^); (c) removal degrees and remaining contents of PFOA (1 μg L^−1^) adsorbed by GAC, PAC, PAF-1, PAF-CF_3_ and PAF-C_2_F_5_ after 9 h (adsorbent dosage: 10 mg L^−1^); (d) a summary of adsorption capacities and rates of reported materials for PFOA removal; (e) removal/recovery degrees of PFOA during regeneration tests (removal experiment: [PAF-CF_3_] = 500 mg L^−1^, [PFOA]_0_ = 200 μg L^−1^, 24 h; recovery experiment: PAF-CF_3_ was soaked in methanol for 24 h); (f) kinetics curves of PFOA (200 μg L^−1^) adsorption on PAF-CF_3_ and PAF-C_2_F_5_ (PAF dosage: 500 mg L^−1^) in the presence of humic acid (20 mg L^−1^).

PAF-CF_3_ and PAF-C_2_F_5_ were applied for PFOA removal in simulated natural contaminated water with low PFOA concentration at 1 μg L^−1^ relevant to the application perspective. The kinetics curve of PAF-CF_3_ (Fig. 4a) shows that the removal degree reaches 93.6% within 3 h with a K_obs_ value of 33.33 g mg^−1^ h^−1^ (Fig. 4b). However, PAF-C_2_F_5_ needs a longer time of 5 h to reach the removal degree of 92.6%. This phenomenon is different from the above observation at 200 μg L^−1^ of PFOA. For more comprehensive understanding, the intraparticle diffusion model is adopted for kinetics analysis of PFOA adsorption, alternative to the pseudo-second-order model. In principle, this model includes three phases of external mass transfer, diffusion within particle and equilibrium. Like K_obs_, another rate constant of k_p_ is calculated to quantify the adsorption kinetics at very low PFOA concentrations. As shown in Supplementary Fig. 17, PAF-CF_3_ and PAF-C_2_F_5_ have different rate constants (k_p1_, k_p2_). The existence of k_p1_ in PAF-C_2_F_5_ translates a resistance for PFOA transfer from solution to particle, which is due to the weaker hydration of particle surface, consistent with the larger water contact angle (Supplementary Fig. 3h). The value of k_p2_ for PAF-C_2_F_5_ is slightly smaller than that of PAF-CF_3_, due to higher hydrophobicity of the inner pores (Supplementary Fig. 3h). The performances of PFOA removal for GAC, PAC and PAF-1 are compared in Fig. 4c. Both PAF-CF_3_ and PAF-C_2_F_5_ exhibit high removal degrees, yielding residual solutions with PFOA concentrations of 37.9 ng L^−1^ and 43.3 ng L^−1^, respectively. The remaining concentrations after adsorption measurements are below the lifetime health advisory level (70 ng L^−1^) set by the U. S. Environment Protection Agency in 2016. Figure 4d summarizes adsorption capacities and rates of all relevant materials for PFOA removal from dilute solutions under similar conditions. We can see that PAF-CF_3_ and PAF-C_2_F_5_ have a good balance between capacity and rate; that is to say, they can quickly and massively remove PFOA from water.

As shown in Fig. 4e, the removal degrees remain above 98.2% during four consecutive adsorption runs. PFOA can be thoroughly recovered by a simple wash with methanol. The adsorption-recovery experiment shows that PAF-CF_3_ has good recyclability. The pore and textural structures after regeneration were also characterized by N_2_ sorption and infrared spectroscopy, respectively (Supplementary Fig. 18 and 9). The IR spectrum, BET surface area (966.1 m^2^ g^−1^) and major pore size (1.1 nm) of the regenerated PAF-CF_3_ are similar to those of the fresh PAF-CF_3_, confirming the structural stability of this material. Moreover, the removal degrees and rates of PAF-CF_3_ and PAF-C_2_F_5_ were not compromised by the presence of humic acid in simulated contaminated water (Fig. 4f). The removal degrees for PAF-CF_3_ and PAF-C_2_F_5_ are almost retained at pH = 3.0, 6.7, 9.0 and in presence of 20 mg L^−1^ NaCl (Supplementary Fig. 20), implying that both PAFs have good tolerability under different water conditions.

## CONCLUSION

In summary, the syntheses of functionalized PAF-CF_3_ and PAF-C_2_F_5_ with high stabilities and large surface areas (972.8 m^2^ g^−1^ and 809.8 m^2^ g^−1^) were achieved using fluorine-containing monomers. Both PAFs had open frameworks owing to the tetrahedral geometry of the monomers, and fluorine groups enabled them to have fluorophilicity. They exhibited combined advantages of high capacity and fast rate during PFOA adsorption in water. Adsorption isotherms revealed that the maximum adsorption capacities of PAF-CF_3_ and PAF-C_2_F_5_ reached 746 mg g^−1^ and 935 mg g^−1^, respectively. Kinetics studies demonstrated that PAF-CF_3_ and PAF-C_2_F_5_ had high removal rates (K_obs_ = 33.33 g mg^−1^ h^−1^ and 24.43 g mg^−1^ h^−1^) for 1 μg L^−1^ PFOA solution. Their outstanding PFOA removal performances can be attributed to unique C–F···F–C interactions and large surface areas. Besides, PAF-CF_3_ and PAF-C_2_F_5_ are capable of removing PFOA in dilute solution, rendering the PFOA concentration (37.9 ng L^−1^) below the EPA health advisory level (70 ng L^−1^). The high efficiency for PFOA removal and good recyclability cast a new light on the importance of fluorine-functionalized PAFs for adsorptive removal of polyfluorinated substances in polluted water.

## Supplementary Material

nwad191_Supplemental_File
