# Supplementary material for: Unique fluorophilic pores engineering within porous aromatic frameworks for trace perfluorooctanoic acid removal
Source: Natl Sci Rev. 2023 Jul 10;10(10):nwad191. doi: 10.1093/nsr/nwad191 (PMC10476896; doi:10.1093/nsr/nwad191)
Supplement: nwad191_Supplemental_File [file nwad191_supplemental_file.pdf]

Supporting Information

Unique fluorophilic pores engineering within porous aromatic frameworks for trace perfluorooctanoic acid removal

Chi Zhang[a],†, Junchao Dong[a],†, Panpan Zhang[a],†, Lei Sun[b], Liu Yang[a], Wenjian Wang[a], Xiaoqin Zou[a],, Yunning Chen[a], Qingkun Shang[a], Danyang Feng[a],* and Guangshan Zhu[a],**

[a] Faculty of Chemistry, Northeast Normal University, Changchun 130024, China

[b] Institute of Molecular Sciences and Engineering, Institute of Frontier and Interdisciplinary Science, Shandong University, Qingdao 266237, China

†Equally contributed to this work, *Corresponding authors.

Ⅰ. Materials and Instrumentation

Materials. The main materials used in this work included: triphenylmethanol (99%, Innochem), 2-bromoaniline (98%, Aladdin), isopentyl nitrite (95%, Innochem), copper(I) iodide (99%, Bidepharm), sodium trifluoroacetate (98%, Innochem), sodium 2,2,3,3,3-pentafluoropropanoate (98%, Leyan), bis(1,5-cyclooctadiene)nickel(0) ($Ni(cod)_2$, 96%, Innochem), 1,5-cyclooctadiene (cod, 99%, Acros), 2,2'-dipyridyl (99%, Aladdin), perfluorooctanoic acid (PFOA, 99.6%, TMstandard), humic acid (HA, fulvic acid ≥90%, Aladdin), phosphinic acid (H_3PO_2, 50 wt% in water, Energy Chemical), N,N-dimethylformamide (DMF, 99.9%, Innochem), 1-methyl-2-pyrrolidinone (NMP, 99.5%, Innochem). Other chemicals and reagents were purchased from domestic suppliers in China and used without purification. Water used in the experiments was all deionized at neutral pH.

Instrumentation. The morphologies of PAF-CF_3 and PAF-C_2F_5 were observed on field emission scanning electron microscopy (FE-SEM) (Hitachi SU-8010). The element contents were measured on an energy-dispersive X-ray spectrometer (EDX) equipped with FE-SEM. Infrared spectra were measured using a Nicolet IS50 Fourier transform infrared spectrometer (FTIR). Thermogravimetric (TG) curves were recorded on a Mettler Toledo TGA/DSC 3+ thermogravimetric analysis system from 303 K to 1073 K at a heating rate of 10 K min^{-1} in air flow. ^1H and ^{19}F NMR spectra of monomers were recorded on a Bruker Avance NEO 500 MHz nuclear magnetic resonance (NMR) spectrometer. ^{13}C and ^{19}F NMR spectra of PAFs were measured on a Bruker Avance Ⅲ 600 MHz solid-state nuclear magnetic resonance (NMR) spectrometer. N_2 sorption experiments at 77 K were carried out on an Autosorb iQ2 adsorptometer, Quantachrome Instrument. Mass spectra (MS) were measured on a gas chromatography-mass spectrometry (TSQ 8000 Evo, Thermo Scientific).

PFOA Contents Measurements. PFOA contents of the water samples were determined by a high-resolution liquid chromatography coupled with a quadrupole/orbitrap mass spectrometer (Q EXACTIVE FOCUS, Thermo Scientific). The mobile phase was 100% acetonitrile with a flow rate of 0.2 mL min^{-1}. The setting parameters for mass spectrometry with electrospray ionization (ESI) operated in negative ion mode and selected ion monitoring mode were listed in the following: sheath gas flow rate 30, aus gas flow rate 15, sweep gas flow rate 0, spray voltage 4.2 keV, capillary temperature of 320 °C and aus gas heater temperature of 300 °C. All subsequent adsorption experiments were repeated in triplicate for minimizing experimental errors.

Calculation Method. The molecular interaction energy was calculated using the program Gaussian16 [1]. The geometry optimizations were performed through the combination of B3LYP functional [2] and D3 version of Grimme's dispersion correction (GD3) [3]. The vibrational frequencies of the optimized configurations were analyzed to validate that the configurations correspond to a local minimum. Two basis sets, 6-31G(d) and 6-31++G(d,p), were used in the geometry optimization and single-point energy calculation, respectively. The interactions between adsorbent and adsorbate were directly obtained from the counterpoise correction calculation.

Ⅱ. Synthetic Procedures

Synthesis of triphenyl(3-bromo-4-aminophenyl)methane

Triphenylmethanol (6.5 g) and 2-bromoaniline (4 mL) in a mixed solution of acetic acid (75 mL) and conc. HCl (12.5 mL) were stirred at 120°C for 6 h. After cooled to room temperature, the reaction system was neutralized by NaOH solution. White crude product was formed, filtrated and purified by recrystallization with CH_2Cl_2 and EtOH. Yield: 69.4%.

Synthesis of 1-bromo-3-tritylbenzene

Triphenyl(3-bromo-4-aminophenyl)methane (1242 mg), conc. H_2SO_4 (1 mL) and EtOH (6 mL) were stirred in a reaction flask. The mixture was cooled to -15 °C, after which isopentyl nitrite (0.7 mL) was added dropwise. The above reaction mixture was stirred for another 1 h under -15 °C. Finally, 50% H_3PO_2 (1.5 mL) was slowly added, and the stirring was carried out overnight at 50 °C. The precipitate was purified by column chromatography using hexane as an eluent. Yield: 60.0%.

Synthesis of 1-trifluoromethyl-3-tritylbenzene

1-Bromo-3-tritylbenzene (800 mg), CF_3COONa (1088 mg) and CuI (762 mg) were suspended in NMP (30 mL). The mixture was heated at 160 °C under nitrogen atmosphere for 24 h. After cooled to room temperature, the reaction mixture was diluted with ethyl acetate and washed with H_2O, and then the solvent of the organic layer was removed under reduced pressure. Pure product was separated and purified by column chromatography using hexane as an eluent. Yield: 96.0%.

Synthesis of (4-bromo-3-trifluoromethylphenyl)tris(4-bromophenyl)methane

Bromine (4.0 mL) was dripped slowly to 1-trifluoromethyl-3-tritylbenzene (2.25 g) while stirring at room temperature. The reaction solution was stirred overnight and then poured into EtOH which was precooled at -78 °C. The crude product was obtained by filtration and then washed with saturated aqueous $NaHSO_3$ solution. Pure product was obtained by column chromatography purification using hexane as an eluent. Yield: 87.1%.

Synthesis of 1-pentafluoroethyl-3-tritylbenzene

1-Pentafluoroethyl-3-tritylbenzene was synthesized by pentafluoroethylation of 1-bromo-3-tritylbenzene similar to 1-trifluoromethyl-3-tritylbenzene, using C_2F_5COONa instead of CF_3COONa. Yield: 53.6%.

Synthesis of (4-bromo-3-pentafluoroethylphenyl)tris(4-bromophenyl)methane

(4-Bromo-3-pentafluoroethylphenyl)tris(4-bromophenyl)methane was synthesized by repeating the procedure of the above-mentioned bromination. Yield: 67.5%.

Synthesis of PAF-CF_3 and PAF-C_2F_5

2.25 g Bis(1,5-cyclooctadiene)nickel(0) ([Ni(cod)$_2$]) and 1.28 g 2,2'-bipyridyl were suspended in 60 mL dehydrated DMF followed by adding 1.05 mL 1,5-cyclooctadiene (cod). After heated at 80 °C for 1 h, the mixture was added by 1.10 g (4-bromo-3-trifluoromethylphenyl)tris(4-bromophenyl)methane or 1.18 g (4-bromo-3-pentafluoroethylphenyl)tris(4-bromophenyl)methane which was separately dissolved in 60 mL dehydrated DMF. The mixture was stirred at this temperature for 3 d. After cooling to room temperature, the reaction system was added by conc. HCl and the resultant precipitate was filtered off and washed with H_2O, $CHCl_3$ and THF by Soxhlet extraction in turn. Pale yellow solid product of PAF-CF_3 or PAF-C_2F_5 was obtained after drying at 80 °C for 12 h in vacuum.

Ⅲ. Batch Adsorption Studies of PFOA and Regeneration

1. Adsorption Isotherms

PAFs (4 mg) were added into the PFOA solutions with concentrations of 10, 20, 30, 50, 100, 200, 400, 600, 800 and 1000 mg L^{-1} respectively and stirred for 48 h. The suspensions were filtered with 0.22 μm PES inorganic syringe filters and the concentrations of filtrates were analyzed by

LC-MS. Taking account of the losses of PFOA, control experiments were performed under the same condition with no adsorbent addition.

Data were fitted by Langmuir and Freundlich adsorption models for analysis.

Langmuir adsorption model:

$$\frac{1}{q_e} = \frac{1}{q_{max,e}} + \frac{1}{q_{max,e}K_L C_e}$$

q_e (mg g^{-1}) is the amount of PFOA adsorbed by adsorbent at each concentration in equilibrated state.

$q_{max,e}$ (mg g^{-1}) is the maximum adsorption capacity of adsorbent at equilibrium.

K_L (L $mmol^{-1}$) is the Langmuir equilibrium constant.

C_e (mmol L^{-1}) is each concentration of PFOA in equilibrated state.

Freundlich adsorption model:

$$q_e = K_F C_e^{\frac{1}{n}}$$

q_e (mg g^{-1}) is the amount of PFOA adsorbed by adsorbent at each concentration in equilibrated state.

K_F (mg g^{-1})(L $mg^{-1})^{1/n}$ is the Freundlich constant.

C_e (mg L^{-1}) is each concentration of PFOA in equilibrated state.

n is an indicator of adsorption strength.

2. Adsorption Kinetics

High Concentration (200 μg L^{-1}): Prior to the adsorption kinetics experiment, 50 mg of PAFs were rehydrated by fully dispersing in 95 mL of deionized H_2O through vigorous sonication and agitation. 5 mL of PFOA solution (4 mg L^{-1}) was added to form an initial PFOA solution of 200 μg L^{-1}. 1 mL of samples were taken at predetermined time (1, 2, 5, 30, 60, 120, 240, 360 and 480

min) and filtered with 0.22 μm PES inorganic syringe filters. Control experiments were carried out under the same condition without adsorbent, giving the losses of PFOA as references. All the actual concentrations of initial PFOA solution and samples were measured by LC-MS.

Low Concentration (1 μg L^{-1}): 1 mg of PAF was added to 100 mL of deionized H_2O followed by vigorous sonication and agitation for rehydration before the adsorption kinetics experiment. 1 mL of PFOA solution (0.1 mg L^{-1}) was added to form an initial PFOA solution of 1 μg L^{-1}. Next steps were the same as that for the adsorption of PFOA solution with high concentration (200 μg L^{-1}), except the sampling time of 1, 5, 30, 60, 180, 300, 420 and 540 min.

The PFOA removal degree (R, %) was determined by the following equation:

$$\mathrm{R}\ (\%) = \frac{C_0 - C_t}{C_0} \times 100\%$$

C_0 (μg L^{-1}) and C_t (μg L^{-1}) are the initial and residual concentrations of PFOA solution.

The amount of PFOA adsorbed at the adsorbent was determined using the following equation:

$$q_t = \frac{C_0 - C_t}{C_A}$$

q_t (mg g^{-1}) is the amount of PFOA adsorbed on per gram of adsorbent at time t (h).

C_0 (μg L^{-1}) is the initial concentration of PFOA solution.

C_t (μg L^{-1}) is the residual concentration of PFOA solution at time t (h).

C_A (mg L^{-1}) is the concentration of adsorbent.

Data were fitted by Ho and McKay's pseudo-second-order adsorption model and intraparticle diffusion model for analysis.

Ho and McKay's pseudo-second-order adsorption model:

$$\frac{t}{q_t} = \frac{t}{q_e} + \frac{1}{K_{obs} {q_e}^2} = \frac{t}{q_e} + \frac{1}{v_0}$$

q_e (mg g^{-1}) is the amount of PFOA adsorbed by adsorbent at equilibrium.

K_{obs} (g mg^{-1} h^{-1}) is the rate constant of pseudo-second-order adsorption.

Intraparticle diffusion model:

$$q_t = k_p t^{1/2} + C$$

k_p (mg g^{-1} h$^{-1/2}$) is the intraparticle diffusion rate constant.

C (mg g^{-1}) is an indicator of the boundary layer effect.

3. Equilibrium Adsorption at Low Concentration

The batch adsorption experiments of PFOA (1 μg L^{-1}) by GAC, PAC, PAF-1, PAF-CF_3 and PAF-C_2F_5 (10 mg L^{-1}) were performed by consulting the same procedures as above. 1 mL of the sample was collected at 9 h and filtered with 0.22 μm PES inorganic syringe filter. The control experiments were carried out under the same condition without addition of adsorbents, giving the losses of PFOA as references. And the blank sample was also taken at 9 h.

4. Regeneration Studies

Adsorption Experiments: 50 mg of PAF-CF_3 were added to a solution of PFOA (100 mL, 200 μg L^{-1}) and kept stirring for 24 h. 1 mL of the suspension was taken at 24 h and then filtered with a 0.22 μm PES inorganic syringe filter, and the concentration of resulting solution was determined by LC-MS. PFOA-loaded PAF was recycled by filtration through a filter membrane.

Recovery Experiments: PFOA-loaded PAF-CF_3 was regenerated by soaking in MeOH (100 mL) and then stirring for 24 h. The suspension was separated by centrifugation to recover the adsorbent. The supernatant was concentrated under vacuum, and the residue was re-dissolved in the deionized water with the same volume of MeOH. The concentration of the recovered PFOA was measured by LC-MS.

Ⅳ. Supporting Figures and Tables

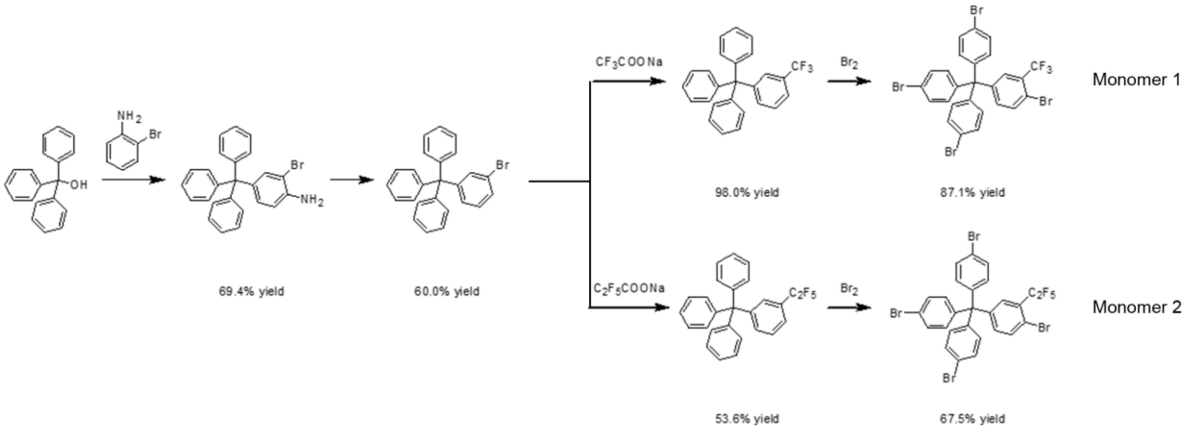

Scheme S1. Synthetic routes of monomer 1 ((4-bromo-3-trifluoromethylphenyl)tris(4-bromophenyl)methane) and monomer 2 ((4-bromo-3-pentafluoroethylphenyl)tris(4-bromophenyl)methane).

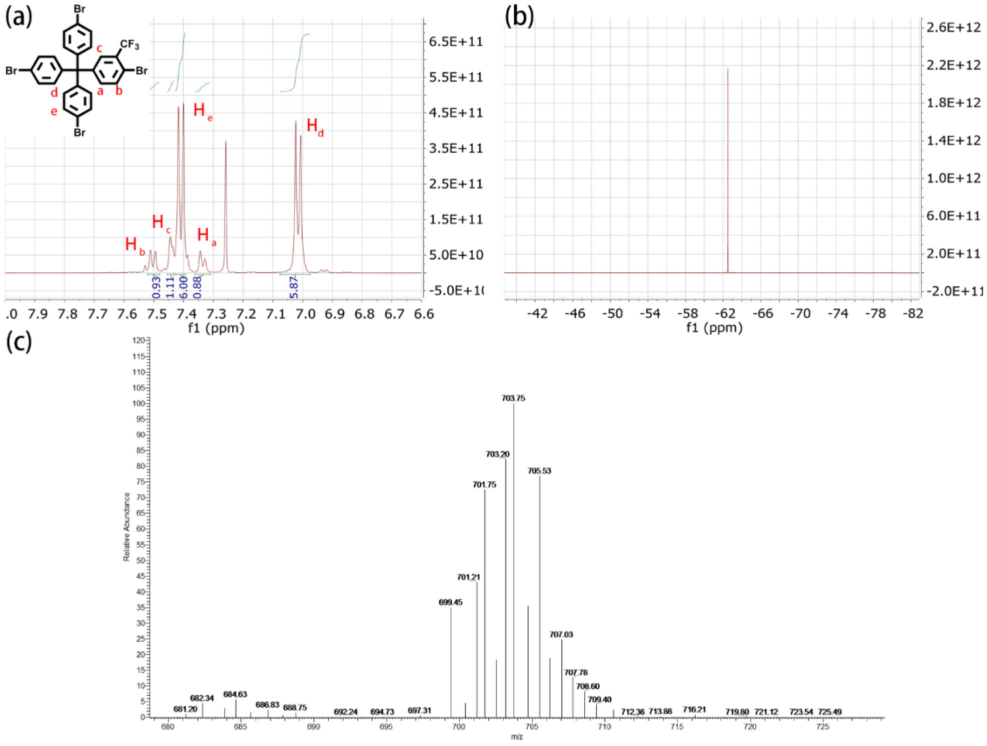

Figure S1. Liquid ^{1}H, ^{19}F NMR spectra (a, b) and mass spectrum (c) of Monomer 1 in specific region.

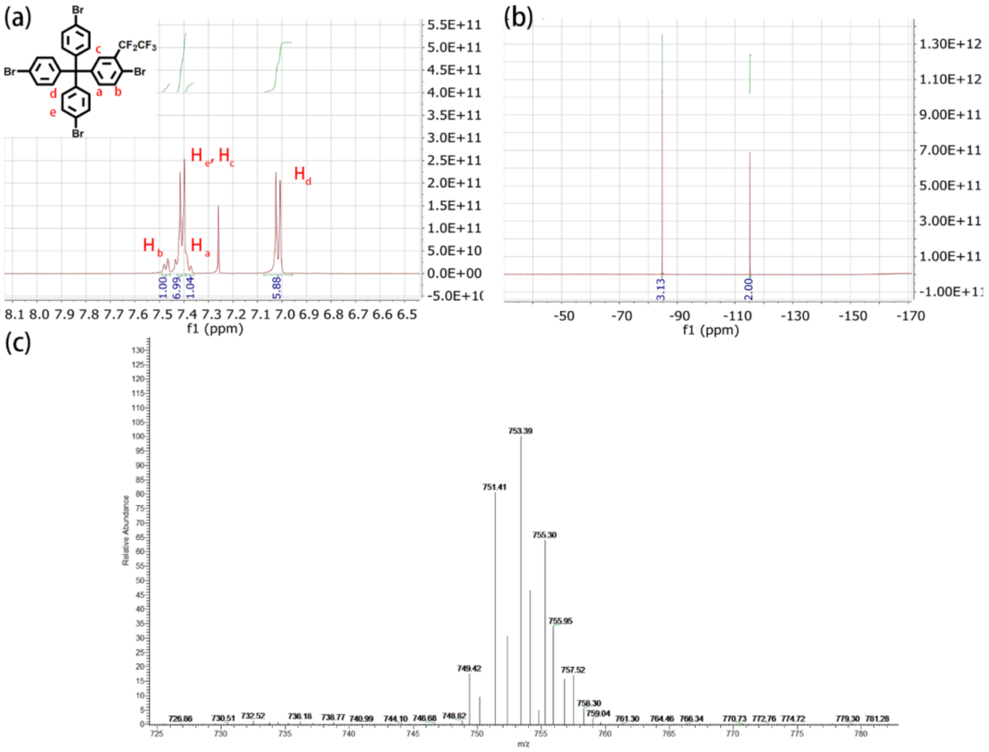

Figure S2. Liquid ^{1}H, ^{19}F NMR spectra (a, b) and mass spectrum (c) of Monomer 2 in specific region.

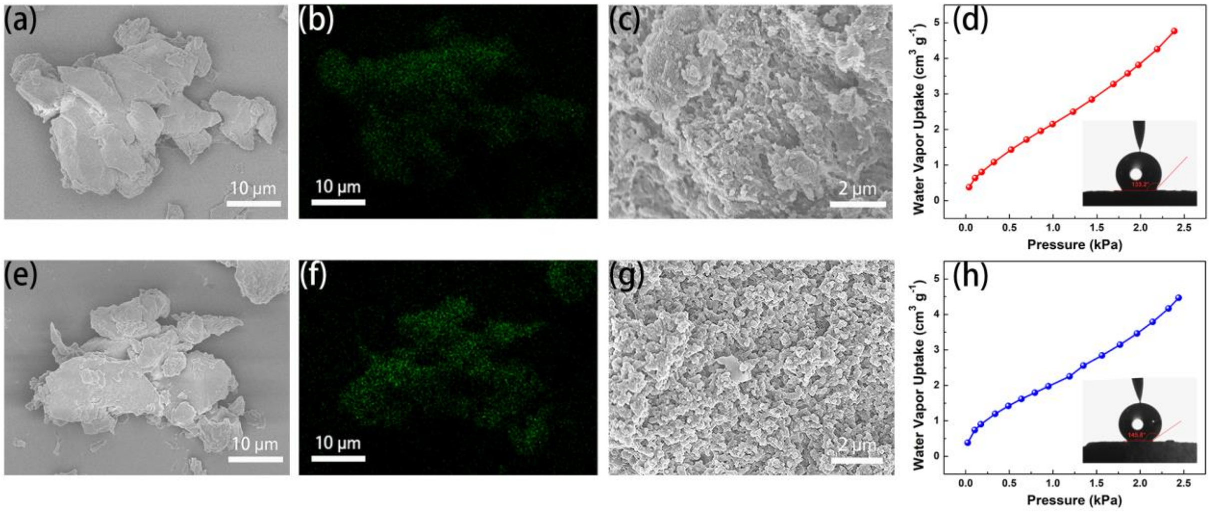

Figure S3. SEM images and corresponding EDX elemental mapping of PAF-CF_3 (a, b) and PAF-C_2F_5 (e, f), the green color represents the fluorine element; (c) and (g) are magnified images

of (a) and (e); (d, h) water adsorption isotherms of PAF-CF_3 and PAF-C_2F_5 at 298 K, insets are water contact angles of PAF-CF_3 and PAF-C_2F_5.

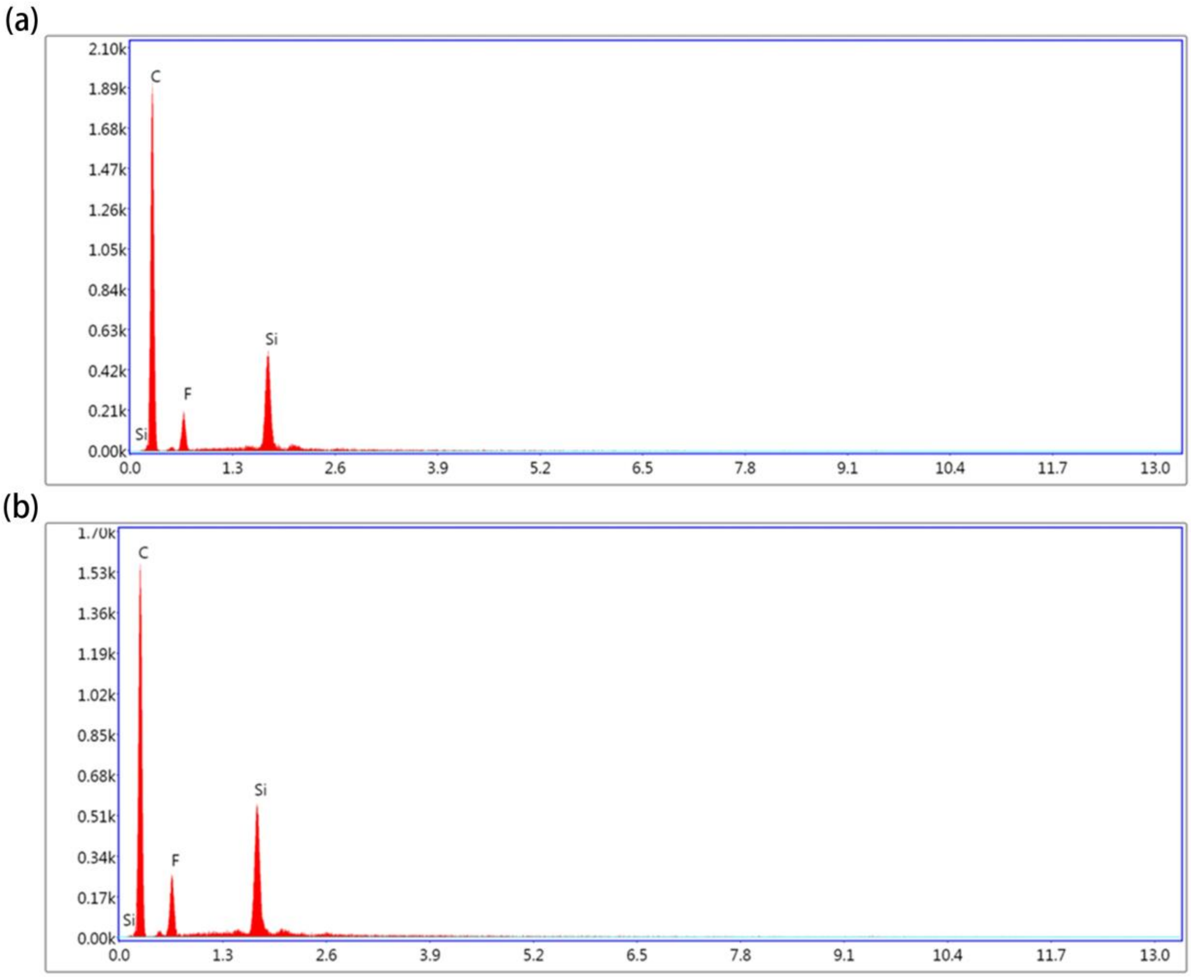

Figure S4. Elemental compositions of (a) PAF-CF_3 and (b) PAF-C_2F_5 given by energy-dispersive X-ray spectra (EDX). The Si signals are coming from the silicon substrates used for the measurements.

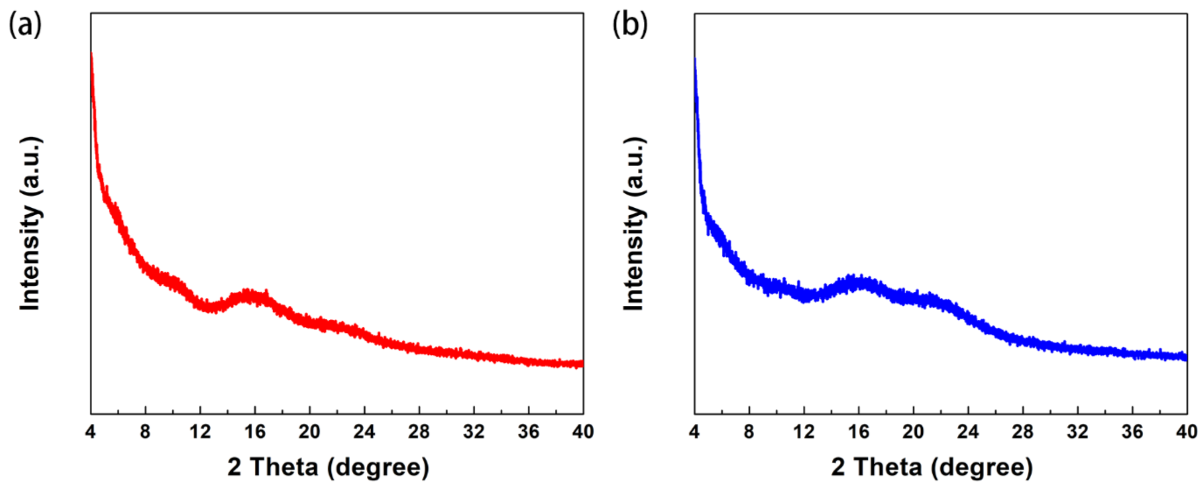

Figure S5. Powder X-ray diffraction patterns of PAF-CF_3 (a) and PAF-C_2F_5 (b).

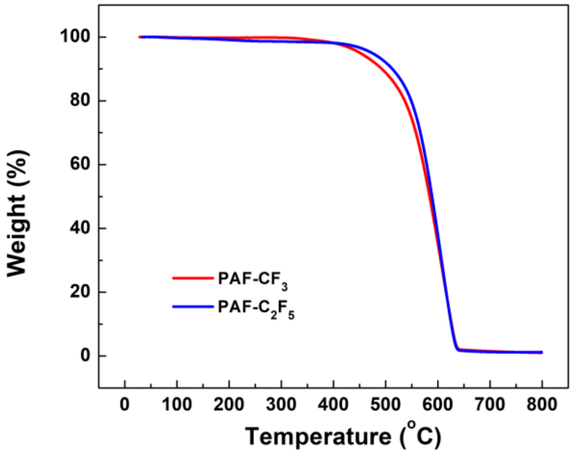

Figure S6. Thermogravimetric (TG) curves of PAF-CF_3 and PAF-C_2F_5.

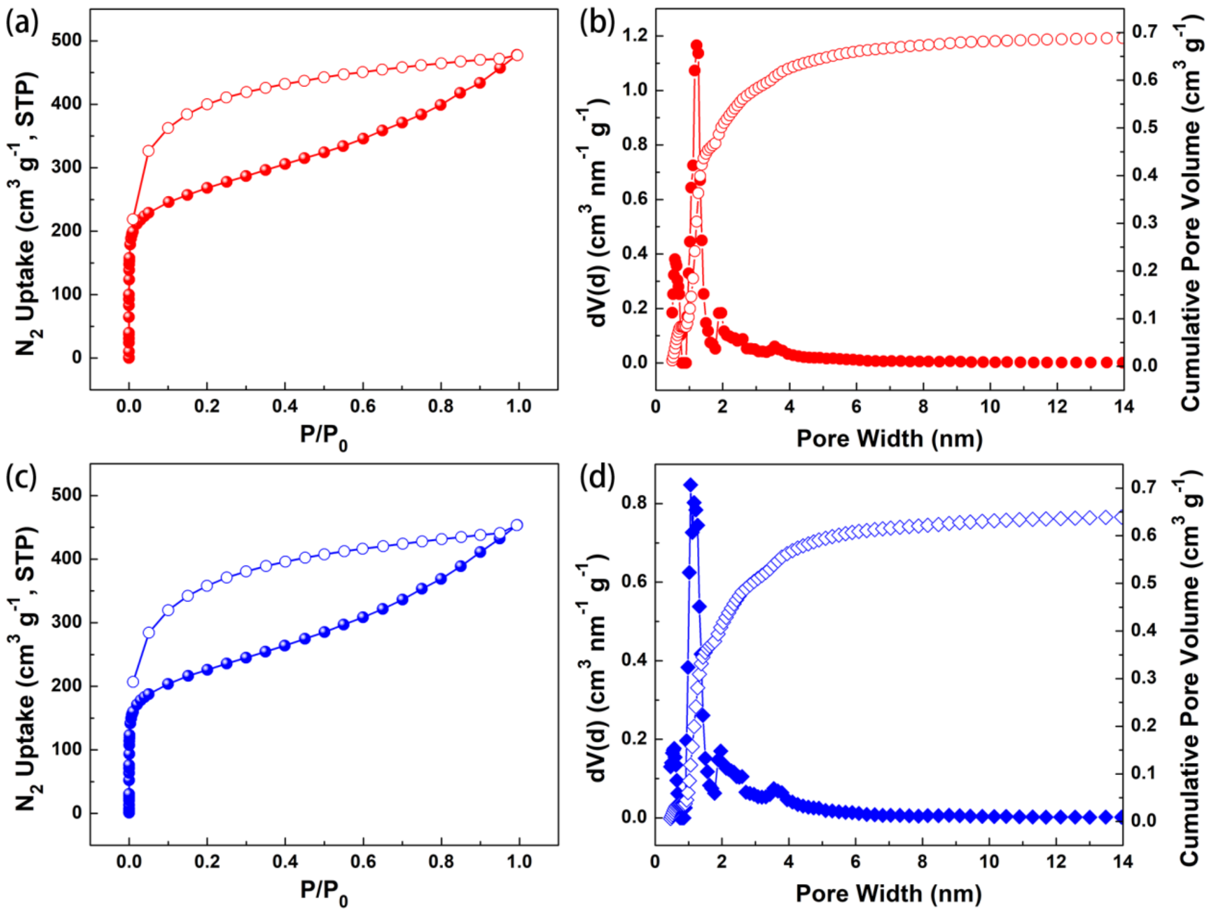

Figure S7. N_2 adsorption-desorption isotherms at 77 K of PAF-CF_3 (a) and PAF-C_2F_5 (c) (filled symbols for adsorption and open symbols for desorption), pore size distributions of PAF-CF_3 (b) and PAF-C_2F_5 (d) calculated by nonlocal density functional theory (filled symbols for differential pore volumes and open symbols for cumulative pore volumes).

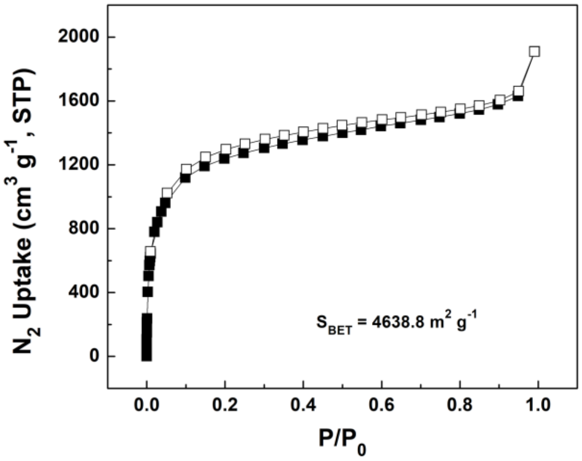

Figure S8. N_2 adsorption-desorption isotherms at 77 K of PAF-1 (filled symbols for adsorption and open symbols for desorption).

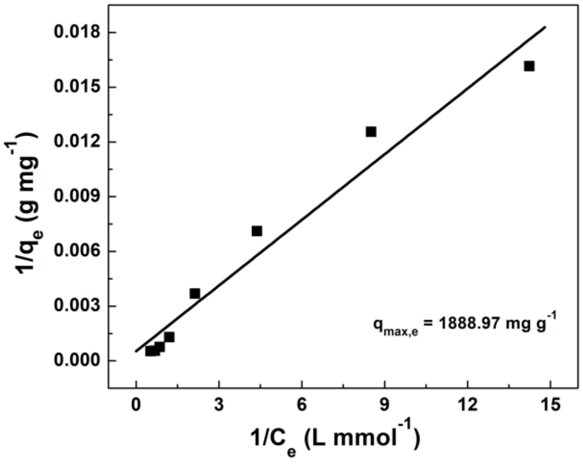

Figure S9. Adsorption isotherm of PFOA on PAF-1 and the Langmuir fit (PAF-1 dosage: 200 mg L^{-1}, $[PFOA]_0$ = 10-1000 mg L^{-1}).

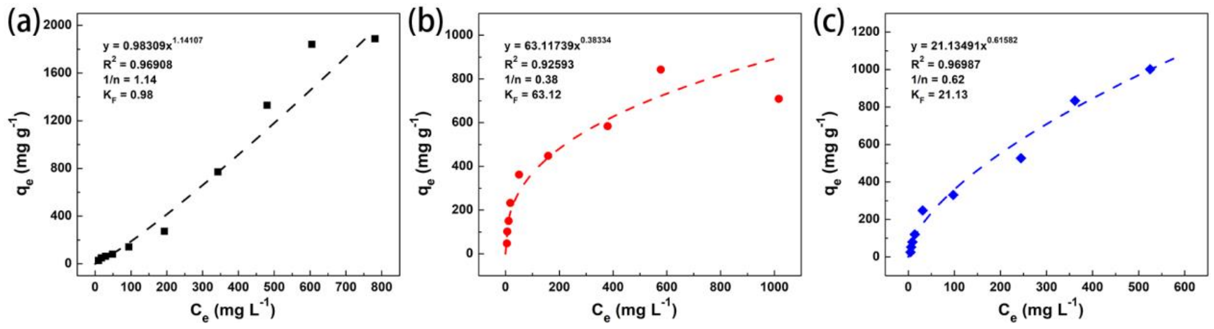

Figure S10. Adsorption isotherms of PFOA on (a) PAF-1, (b) PAF-CF_3 and (c) PAF-C_2F_5 fitted to Freundlich model (PAF dosage: 200 mg L^{-1}, $[PFOA]_0$ = 10-1000 mg L^{-1}).

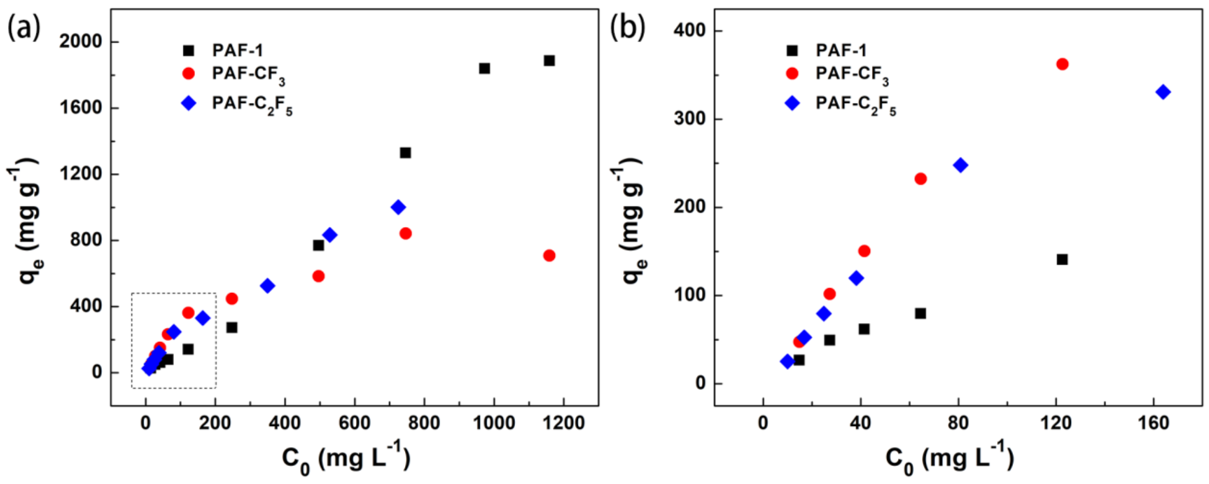

Figure S11. (a) The variations of PFOA adsorbed amounts at equilibrium in function of initial PFOA concentration; (b) the enlarged view of the region icon with a dotted box in (a).

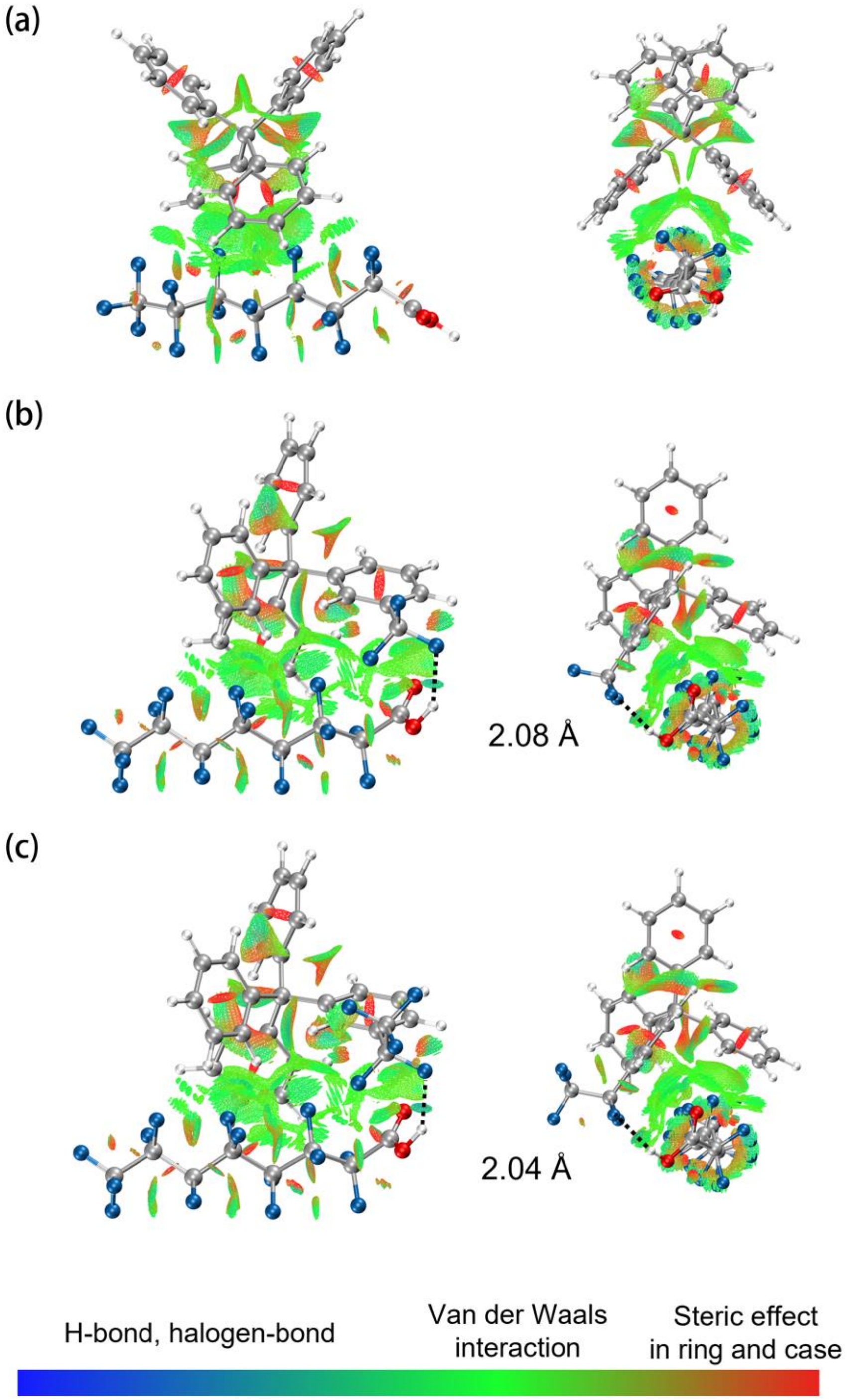

Figure S12. Isosurface of the interaction region indicator (IRI) for (a) PAF-1, (b) PAF-CF_3, (c) PAF-C_2F_5 and PFOA from two directions. The isosurface was mapped according to the function IRI = 1.0 $sign(\lambda_2)\rho$.

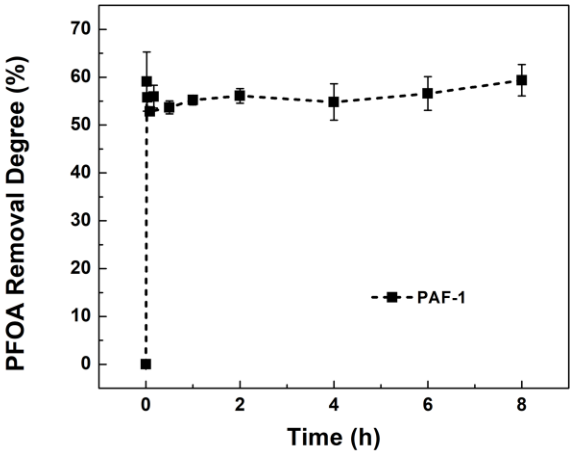

Figure S13. Kinetics curve of PFOA (200 μg L^{-1}) adsorption on PAF-1 (PAF-1 dosage: 500 mg L^{-1}).

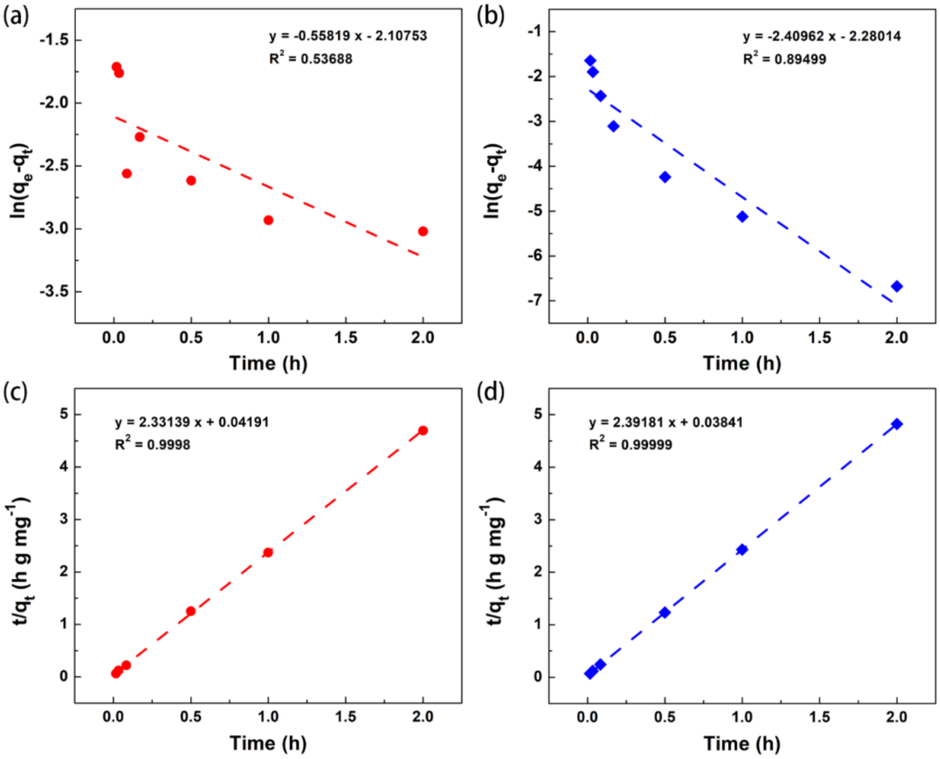

Figure S14. Pseudo-first-order (a, b) and pseudo-second-order (c, d) plots of PFOA (200 μg L^{-1}) on PAF-CF_3 (a, c) and PAF-C_2F_5 (b, d) (PAF dosage: 500 mg L^{-1}). The pseudo-first-order model is described as $\ln(q_e - q_t) = \ln q_e - k_1 t$, k_1 (h^{-1}) is the adsorption rate constant.

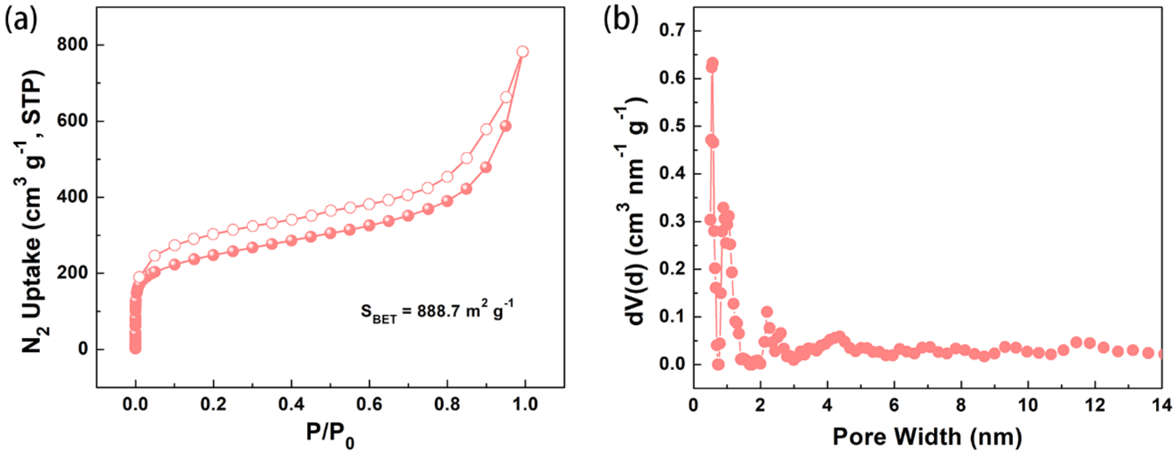

Figure S15. (a) N_2 adsorption-desorption isotherms at 77 K (filled symbols for adsorption and open symbols for desorption) of PAF-CF_3 after PFOA adsorption; (b) the pore size distribution of PAF-CF_3 after PFOA adsorption calculated by nonlocal density functional theory.

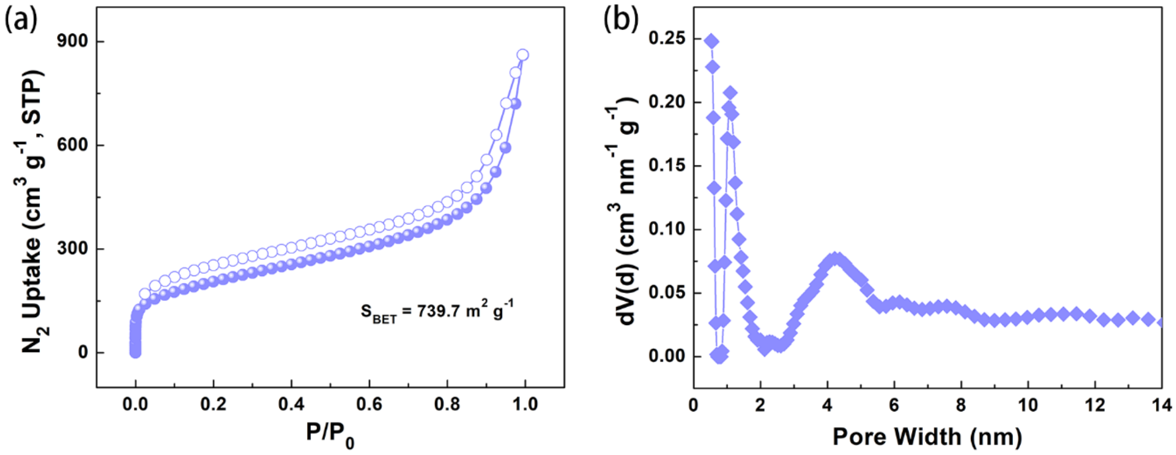

Figure S16. (a) N_2 adsorption-desorption isotherms at 77 K (filled symbols for adsorption and open symbols for desorption) of PAF-C_2F_5 after PFOA adsorption; (b) the pore size distribution of PAF-C_2F_5 after PFOA adsorption calculated by nonlocal density functional theory.

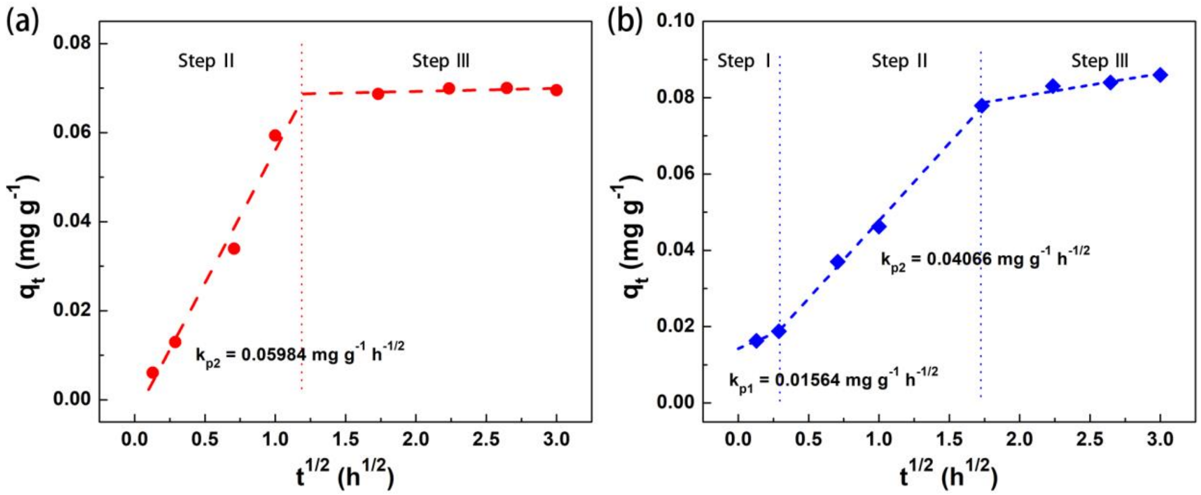

Figure S17. Intraparticle diffusion model fits of PFOA (1 μg L^{-1}) on PAF-CF_3 (a) and PAF-C_2F_5 (b) (PAF dosage: 10 mg L^{-1}). Note: step Ⅰ is not observed in the adsorption process of PAF-CF_3 due to the rapid PFOA transfer in the external process of solution to particle, and thus k_{p1} is not measured.

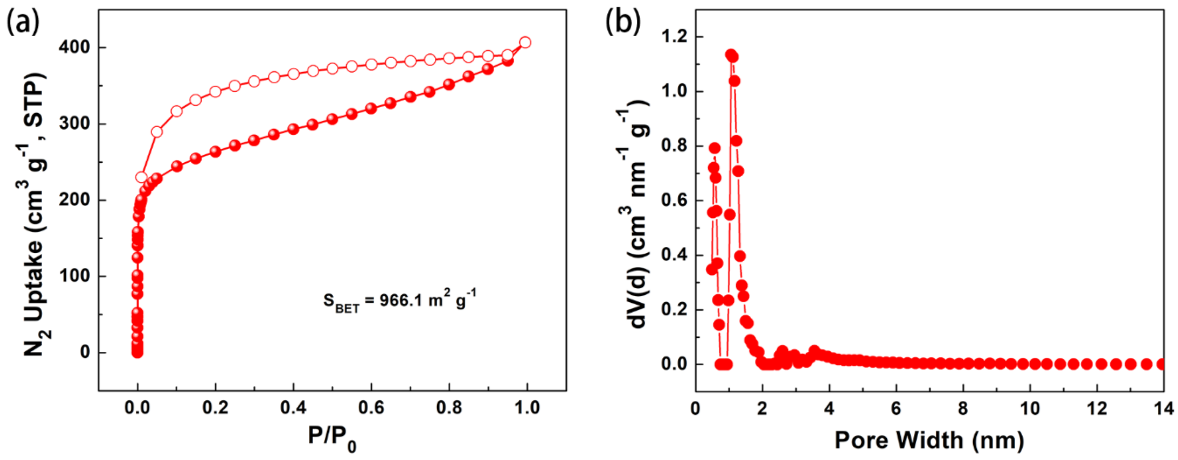

Figure S18. (a) N_2 adsorption-desorption isotherms at 77 K (filled symbols for adsorption and open symbols for desorption) of the regenerated PAF-CF_3; (b) the pore size distribution of the regenerated PAF-CF_3 calculated by nonlocal density functional theory.

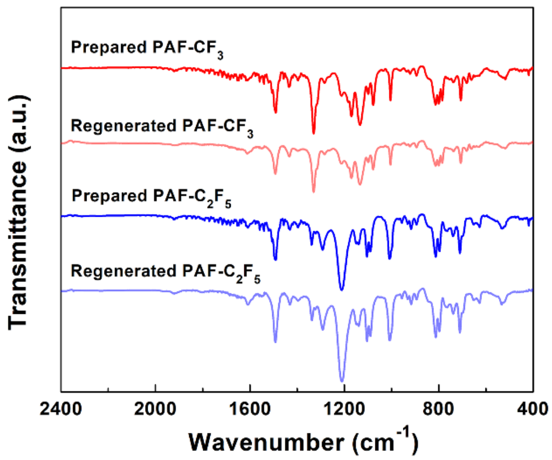

Figure S19. Infrared (IR) spectra of fresh prepared and regenerated PAF-CF_3 and PAF-C_2F_5.

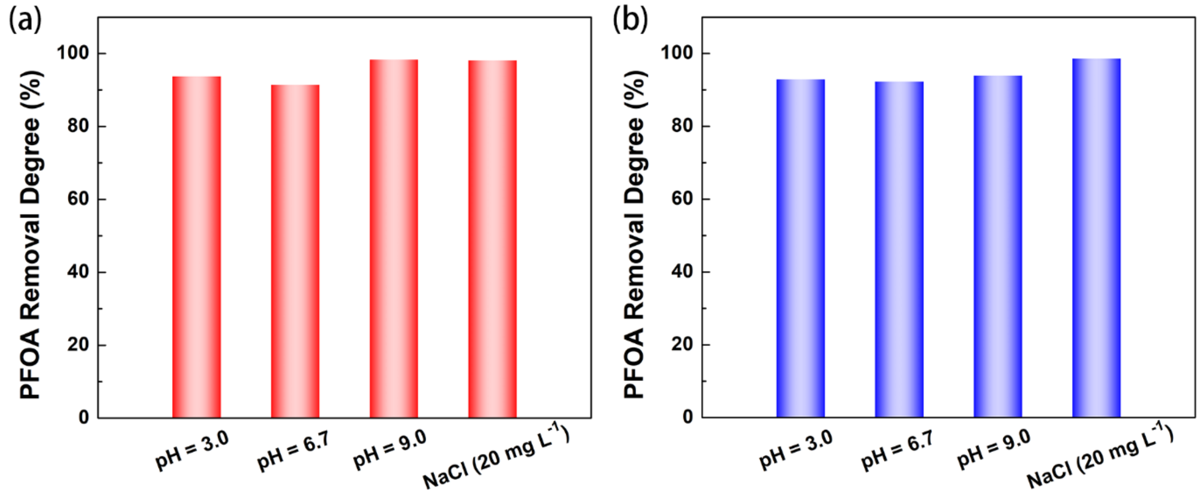

Figure S20. Removal degrees of PFOA (200 μg L^{-1}) adsorbed by PAF-CF_3 (a) and PAF-C_2F_5 (b) (PAF dosage: 500 mg L^{-1}) in different water environments.

Table S1. Element contents of carbon and fluorine in PAF-CF_3 and PAF-C_2F_5 determined by EDX.

Material	Element Content (%)		C: F (atomic ratio)	
	C	F	Theoretical	Experimental
**PAF-CF_3**	89.57	10.43	8.67	8.59
**PAF-C_2F_5**	86.61	13.39	5.4	6.47

Table S2. Langmuir and Freundlich parameters for the adsorption of PFOA on different adsorbents.

Adsorbent	S_{BET} (m^2 g^{-1})	Langmuir Fit		Freundlich Fit		Reference
		$q_{max,e}$ (mg g^{-1})	K_L (L $mmol^{-1}$)	K_F	n	
**Fe_3O_4@GAC(2:1)**	797	592.53	7.852	1.528[a)]	2.74	[4]
**GAC**	955	460.03	7.318	1.079[a)]	3.26	
**GAC**	712	161.49	18	0.47[a)]	3.57	[5]
**PAC**	812	277.43	59	0.83[a)]	5	
**AI400**	---	1209.08	69	3.35[a)]	7.69	
**PACFs**	1782	302.27	77.07	0.8[a)]	7.14	[6]
**PAC**	1227	202.89	84.99	0.53[a)]	9.09	
**GAC**	815	178.05	39.78	0.46[a)]	6.25	
**Hydrotalcite**	200	2567.23	6.75	27.04[a)]	1.06	[7]
**PAC**	500-2000	426.49	20.8	1.35[a)]	2.1	
**MIL-101(Cr)**	2560	459.62	8.08	1.26[a)]	2.44	[8]
**MIL-101(Cr)-NH_2**	1195	289.85	3.90	0.63[a)]	2.13	

**MIL-101(Cr)-NMe$_3$**	445	492.74	20.27	1.24$^{a)}$	6.25	
**MIL-101(Cr)-DMEN**	1692	534.15	7.66	1.53$^{a)}$	2.27	
**MIL-101(Cr)-QDMEN**	1530	753.61	9.41	2.01$^{a)}$	2.56	
**DFB-CDP**	7.29	34	220	12.0$^{b)}$	2.4	[9]
**PAC**	980	434.8	53.83	76.81$^{b)}$	2.5	[10]
**HMS**	712	7.8	2.48	0.07$^{b)}$	0.87	
**A-HMS**	262	69.9	9.11	1.40$^{b)}$	1.22	
**M-HMS**	912	48.1	5.38	0.64$^{b)}$	1.22	
**OD-HMS**	476	64.5	8.28	0.83$^{b)}$	1.17	
**Ti-HMS**	766	76.9	4.55	0.03$^{b)}$	0.66	
**NaY**	653	24.0	9.52	2.09$^{b)}$	2.11	
**HY**	370	33.3	4.97	0.40$^{b)}$	1.23	
**PEI-*f*-CMC**	7.8	2.32	47700.9	11.46$^{b)}$	0.51	[11]
**CHMA-600**	106.3	2842.6	0.56	79.5$^{b)}$	0.353	[12]
**MWCNTs@MIPs**	82.4	12.4	2380.90	5.65$^{b)}$	0.273	[13]
**MWCNTs@NIPs**	78.5	7.31	261.69	1.89$^{b)}$	0.462	
**Fe-BTC**	1051	548.2	2.60	19.0$^{b)}$	2	[14]
**MIL-100-Fe**	1237	426.6	2.32	14.8$^{b)}$	2.05	
**MIL-101-Fe**	1811	490.1	2.31	16.5$^{b)}$	2.02	
**HWC biochar**	453	31.7	128.36	8.5$^{b)}$	3.125	[15]
**PWC biochar**	413	27.7	215.32	8.7$^{b)}$	3.448	
**AGO aerogel**	---	1343	3.44	40.8$^{b)}$	1.84	[16]
**3D SG-TiO$_2$ QDa**	---	26.17	53.42	80.33$^{b)}$	2.55	[17]
**FCX4-P**	417	188.7	---	---	---	[18]
**FCX4-BP**	451	169.5	---	---	---	

**PAC**	1000	16.502	250.93	24.238[b]	2.22	[19]
**GAC**	534	52.8	8281.4	2.42[c]	1.12	[20]
**Montmorillonite**	67.52	0.108	2567.23	1.88[d]	1.61	[21]
**Kaolinite**	23.11	0.104	2401.61	1.70[d]	1.61	
**Hematite**	9.9	0.112	1821.91	1.11[d]	1.43	
**Powder MWNTs**	519.7	2.69	526	0.018[a]	3.70	[22]
**MWNTs at 0.6 V**	519.7	405.79	286	6.5[a]	2.08	
**PAF-1**	4638.8	1888.97	0.44	0.98[b]	0.88	This work
**PAF-CF_3**	972.8	746.27	9.43	63.12[b]	2.63	
**PAF-C_2F_5**	809.8	934.58	4.06	21.13[b]	1.61	

a) (mmol g^{-1})(L $mmol^{-1}$)$^{1/n}$; b) (mg g^{-1})(L mg^{-1})$^{1/n}$; c) (μmol g^{-1})(L μmol^{-1})$^{1/n}$; d) (μg g^{-1})(L μg^{-1})$^{1/n}$.

Table S3. Comparison of adsorption kinetics parameters by different adsorbents reported in the literature.

**Adsorbent**	**Kinetics**			**Reference**
	**[Adsorbent] (mg L^{-1})**	**$[PFOA]_0$ (mg L^{-1})**	**K_{obs} (g mg^{-1} h^{-1})**	
**PAF-CF_3**	10	0.001	33.33	This work
	500	0.2	129.7	
**PAF-C_2F_5**	10	0.001	24.43	This work
	500	0.2	148.9	
**DFB-CDP**	10	0.001	2.88	[9]
	400	0.2	64.8	
**PEI-*f*-CMC**	50	0.1	12.78	[11]
	25	0.001	0.06	
**MWCNTs@MIPs**	666.7	0.5	49.26	[13]
**HWC biochar**	333.3	0.001	4.17	[15]

**PWC biochar**	333.3	0.001	50	
**AGO aerogel**	---	10	0.1047	[16]
**3D SG-TiO_2 QDa**	20	5	0.399	[17]
**FCX4-P**	33.3	0.001	3.8	[18]
**PAC**	5000	5	20.88	[19]
**GAC**	200	10	4.72	[20]
**Montmorillonite**	400	0.6	551.3	[21]
**Kaolinite**	400	0.6	131.2	
**Hematite**	400	0.6	431.8	
**Powder MWNTs**	50	0.1	0.32	[22]
**MWNTs at 0.6 V**	50	0.1	3.12	

Table S4. Pore structure data of fresh PAFs, PAFs after PFOA adsorption and recycled PAF-CF_3.

	**Pore size (nm)**	**S_{BET} (m^2 g^{-1})**	**V_{micro} (cm^3 g^{-1})**
**PAF-CF_3**	1.2	972.8	0.51
**PAF-C_2F_5**	1.0	809.8	0.42
**PFOA@PAF-CF_3**	0.57	888.7	0.20
**PFOA@PAF-C_2F_5**	0.52	739.7	0.14
**Recycled PAF-CF_3**	1.1	966.1	0.49

Ⅴ. Supplementary References

1. Frisch MJ, Trucks GW and Schlegel HB *et al*. Gaussian 16 Rev. A.03, Wallingford, CT, 2016.

2. Becke AD. Density-functional thermochemistry. III. The role of exact exchange. *J Chem Phys* 1993; **98**: 5648.

3. Grimme S, Antony J and Ehrlich S *et al*. A consistent and accurate *ab initio* parametrization of density functional dispersion correction (DFT-D) for the 94 elements H-Pu. *J Chem Phys* 2010; **132:** 154104.

4. Xu JH, Liu ZW and Zhao DY *et al*. Enhanced adsorption of perfluorooctanoic acid (PFOA) from water by granular activated carbon supported magnetite nanoparticles. *Sci Total Environ* 2020; **723**: 137757.

5. Yu Q, Zhang RQ and Deng SB *et al*. Sorption of perfluorooctane sulfonate and perfluorooctanoate on activated carbons and resin: Kinetic and isotherm study. *Water Res* 2009; **43**: 1150-8.

6. Chen W, Zhang XP and Mamadiev M *et al*. Sorption of perfluorooctane sulfonate and perfluorooctanoate on polyacrylonitrile fiber-derived activated carbon fibers: in comparison with activated carbon. *RSC Adv* 2017; **7**: 927-38.

7. Rattanaoudom R, Visvanathan C and Boontanon SK. Removal of concentrated PFOS and PFOA in synthetic industrial wastewater by powder activated carbon and hydrotalcite. *J Water Sustain* 2012; **2**: 245-58.

8. Liu K, Zhang SY and Hu XY *et al*. Understanding the adsorption of PFOA on MIL-101(Cr)-based anionic-exchange metal–organic frameworks: Comparing DFT calculations with aqueous sorption experiments. *Environ Sci Technol* 2015; **49**: 8657-65.

9. Xiao LL, Ling YH and Alsbaiee A *et al*. β-Cyclodextrin polymer network sequesters perfluorooctanoic acid at environmentally relevant concentrations. *J Am Chem Soc* 2017; **139**: 7689-92.

10. Punyapalakul P, Suksomboon K and Prarat P *et al*. Effects of surface functional groups and porous structures on adsorption and recovery of perfluorinated compounds by inorganic porous silicas. *Sep Sci Technol* 2013; **48**: 775-88.

11. Ateia M, Attia MF and Maroli A *et al*. Rapid removal of poly- and perfluorinated alkyl substances by poly(ethylenimine)-functionalized cellulose microcrystals at environmentally relevant conditions. *Environ Sci Technol Lett* 2018; **5**: 764-9.

12. Yang YQ, Yang MH and Zheng ZH *et al*. Highly effective adsorption removal of perfluorooctanoic acid (PFOA) from aqueous solution using calcined layer-like Mg-Al hydrotalcites nanosheets. *Environ Sci Pollut Res* 2020; **27**: 13396-408.

13. Cao FM, Wang L and Yao YM *et al*. Synthesis and application of a highly selective molecularly imprinted adsorbent based on multi-walled carbon nanotubes for selective removal of perfluorooctanoic acid. *Environ Sci Water Res Technol* 2018; **4**: 689-700.

14. Yang YQ, Zheng ZH and Ji WQ *et al*. Insights to perfluorooctanoic acid adsorption micro-mechanism over Fe-based metal organic frameworks: Combining computational calculation with response surface methodology. *J Hazard Mater* 2020; **395**: 122686.

15. Inyang M and Dickenson ERV. The use of carbon adsorbents for the removal of perfluoroalkyl acids from potable reuse systems. *Chemosphere* 2017; **184**: 168-75.

16. Tian DY, Geng D and Mehler WT *et al*. Removal of perfluorooctanoic acid (PFOA) from aqueous solution by amino-functionalized graphene oxide (AGO) aerogels: Influencing factors, kinetics, isotherms, and thermodynamic studies. *Sci Total Environ* 2021; **783**: 147041.

17. Zhu C, Xu JL and Song S *et al*. TiO_2 quantum dots loaded sulfonated graphene aerogel for effective adsorption-photocatalysis of PFOA. *Sci Total Environ* 2020; **698**: 134275.

18. Shetty D, Jahović I and Skorjanc T *et al*. Rapid and efficient removal of perfluorooctanoic acid from water with fluorine-rich calixarene-based porous polymers. *ACS Appl Mater Interfaces* 2020; **12**: 43160-6.

19. Qu Y, Zhang CJ and Li F *et al*. Equilibrium and kinetics study on the adsorption of perfluorooctanoic acid from aqueous solution onto powdered activated carbon. *J Hazard Mater* 2009; **169**: 146-52.

20. Zhang D, Luo Q and Gao B *et al*. Sorption of perfluorooctanoic acid, perfluorooctane sulfonate and perfluoroheptanoic acid on granular activated carbon. *Chemosphere* 2016; **144**: 2336-42.

21. Zhao LX, Bian JN and Zhang YH *et al*. Comparison of the sorption behaviors and mechanisms of perfluorosulfonates and perfluorocarboxylic acids on three kinds of clay minerals. *Chemosphere* 2014; **114**: 51-8.

22. Li XN, Chen S and Quan X *et al*. Enhanced adsorption of PFOA and PFOS on multiwalled carbon nanotubes under electrochemical assistance. *Environ Sci Technol* 2011; **45**: 8498-505.
